# The impact of lying about a traumatic virtual reality experience on memory

**DOI:** 10.3758/s13421-018-0885-6

**Published:** 2018-12-19

**Authors:** Tameka Romeo, Henry Otgaar, Tom Smeets, Sara Landstrom, Didi Boerboom

**Affiliations:** 1 Faculty of Psychology and Neuroscience, Forensic Psychology Section, Maastricht University, Maastricht, the Netherlands; 2 Department of Psychology, Gothenburg University, Gothenburg, Sweden; 3 Leuven Institute of Criminology, Catholic University of Leuven, Leuven, Belgium

**Keywords:** Memory, Denial, Fabrication, Coping

## Abstract

The goal of the present experiment was to examine the effect of certain (deceptive) strategies (e.g., false denial) on memory. Specifically, participants were shown a traumatic virtual reality (VR) video of an airplane crash. Following this, participants (*N*= 94) received questions concerning details from the VR scene in a baseline memory task. Then, participants could choose from 3 options how to cope in response to having experienced the VR scene: tell the truth, falsely deny, or fabricate. The majority opted to tell the truth (*n* = 81). A subsample of truth tellers were instructed to falsely deny having seen certain details. One week later, all participants received a source monitoring task in which they were asked (1) whether they remembered talking about these details during an interview, and (2) whether they remembered seeing certain details during the VR experience the week before. Participants had to tell the truth during this task. Participants who were instructed to falsely deny showed impaired memory for presented details that had previously been discussed (i.e., denial-induced forgetting) *and* seen in the VR scene. Also, the presentation of certain details in the baseline memory task seemed to inoculate participants who were instructed to falsely deny from experiencing memory impairment. The current experiment suggests that false denials can have adverse ramifications for memory for what is discussed *and* seen.

Sometimes people, despite their best intentions to be honest, choose to or are forced to lie. In the current experiment, we focused on two types of lies: false denials of factual information and using fabricated details to embellish a story. Numerous motivations can precede the decision to engage in deceitful behavior. For instance, a certain situation may make dishonesty appear to be the more alluring, feasible, or safe option. Such as, for example, the case when people tell lies in order to avoid conflict or spare the feelings of others (DePaulo, Kashy, Kirkendol, Wyer, & Epstein, 1996). Whatever reason, it is understandable that people may opt to falsely deny victimization (e.g., sexual abuse) and guilt (e.g., sexual offending) or fabricate information when placed in high-stakes situations such as those where allegations of child sexual abuse (CSA) are made.

For victims in CSA cases, (false) denial can serve as a coping tool since there may be various perceived advantages to lying about event related information. Feeling guilty, ashamed or responsible (Magnusson, Ernberg, & Landström, 2017; Paine & Hansen, 2002), CSA victims may falsely deny that the abuse occurred or fabricate an alternative explanation (e.g., “My genitals hurt because I fell off my bike”) when they are questioned. An important feature of false denial is that the person is fully aware that they experienced a certain event, but deny it nonetheless. However, some victims may choose to be forthcoming and tell the truth at first, but subsequently change their story because of external factors (Otgaar, Howe, Smeets, & Wang, 2016).

An example of an external motivation to lie is when perpetrators coach (Lyon, Malloy, Quas, & Talwar, 2008), bribe, or threaten victims to give dishonest statements (Paine & Hansen, 2002). A person may also fabricate information by giving false self-generated details or stories, but as is the case with false denial, there is also limited knowledge about the impact of fabrication on memory. In cases where victims are forced to lie (e.g., falsely deny, fabricate), the question that arises is how such lies can have an impact on the memory for an event (e.g., abuse) when telling the truth later on.

To date, research on the memory effects of false denial has used stimuli such as videos (Otgaar, Howe, Memon, & Wang, 2014; Otgaar, Romeo, Howe, & Ramakers, 2018) and pictures (Otgaar et al., 2016). Since real-world generalizability is limited when using stimuli such as videos or pictures, in the current experiment participants experienced a more ecologically valid stimulus (i.e., traumatic virtual reality scene). The chief aim was to investigate memory effects in persons who wish to tell the truth but are instructed to use an alternative cognitive strategy that they did not initially choose (i.e., truth tellers who were instructed to false deny). As research on the effects of fabrication on memory is also quite limited, we also intended to examine the memory effect of this type of lie.

## Coping with adverse experiences

Coping with adverse experiences serves to control the meaning and emotional effects of unfavorable experiences (Lazarus, 1991). When choosing how to cope with an experience, a process of appraisal of the level of threat (i.e., severity and controllability of the stressor; Blaxton & Bergeman, 2017) posed is first undertaken. Ultimately, if a person determines that their resources cannot sufficiently satisfy the internal or external demands of a situation, he or she may use either a cognitive or behavioral strategy in order to cope (Lazarus, 1991). Denial is regarded as one of those coping strategies. The general consensus is that strategies such as self-blame (Daigneault, Hébert, & Tourigny, 2006; Skinner, Edge, Altman, & Sherwood, 2003), avoidance and denial (Guerra, Pereda, Guilera, Abad, 2016; Lazarus, 1991) are inefficient coping strategies. For example, such strategies have been found to be significant predictors in the development of posttraumatic stress disorder (PTSD) in trauma survivors (Hooberman, Rosenfeld, Rasmussen, & Keller, 2010).

Another strategy that can be used to cope with adverse experiences is fabrication. In order to cope, people sometimes integrate fabricated details during the process of cognitively restructuring their perception of an experienced event (Mrazek & Mrazek, 1987). Cognitive restructuring has been found to facilitate resilience in some victims, albeit harmful in the long run. An example of this is when victims reconstruct the motive of a person who has harmed them to make it seem positive. The unabated act of altering the perception of an experience can result in a disconnection from reality (Mrazek & Mrazek, 1987). In doing so, a person can alter details (e.g., add or change details to diminish the impact of a traumatic experience) about an experienced or witnessed event. Although research is scarce concerning the effects of fabrication on memory, there is work on forced confabulation on memory. Forced confabulation actually entails self-generating information (i.e., fabrication), and it has been found that forced confabulation of details results in people recalling false memories (i.e., commission errors). Ackil and Zaragoza (1998) compared the levels of false memories that were generated in young children (i.e., first, third and fourth graders) and college students. All participants viewed a clip from a film and were then asked five true-event and three false-event questions about the film. Participants in the forced confabulation condition were asked to answer all of the questions and to guess even if they did not know. Participants in the control group were required respond to all questions but only if they were certain of the answers and were instructed to not guess. One week later, all of the participants were given a source-monitoring task. Despite knowing that they had confabulated information, participants in all age groups of the confabulation condition reported false memories. Similar results of forced confabulation resulting in false memories were found in more recent studies (Ackil & Zaragoza, 2011; Otgaar et al., 2014; Zaragoza, Payment, Ackil, Drivdahl, & Beck, 2001). Although the work on forced confabulation is related to the current experiment, this work has not been couched in terms of the effects of coping or deceptive strategies on memory. We were therefore interested in understanding how this type of deception could affect memory.

## Coping and memory

Denial has often been regarded as a strategy that might impede the recollection of an event (Baumeister, Dale, & Sommer, 1998). In fact, a recent theoretical memory and deception framework (MAD) postulates that different types of lies can exert a different effect on memory (Otgaar & Baker, 2018). Deception requires the use of more cognitive resources than being honest (Suchotzki, Crombez, Smulders, Meijer, & Verschuere, 2015; Vrij & Fisher, 2016). According to the MAD framework, such false denials will lead to a lack of rehearsal of the event and to forgetting of the lied-upon event. In contrast, when a deceptive strategy such as fabrication is employed, new details are constucted which might become misremembered at a later moment.

Research on the extent to which different cognitive strategies hinder memory processes varies depending on the particular deceptive strategy of focus. For example, Vieira and Lane (2013) demonstrated that denial can impair memory. In their study, participants were first asked to study a series of pictures (e.g., an apple). Next, they were shown items that included both the old and new pictures, and they were instructed to tell the truth, deny, or describe (fabricate) information. Reassessment of participants’ memory 48 hours later showed that in contrast to describing details that they did not see, the denial of details that they had actually seen was associated with less recall of studied items from the first session.

Recently, it has also been shown that (false) denials can uniquely affect memory in that they impair memory for what was discussed with an experimenter. This memory impairment effect is known as *denial-induced forgetting* (DIF). Otgaar et al. (2014) observed DIF in their study in which participants (children and adults) first viewed a video of a theft and were subsequently instructed to falsely deny information. Specifically, in Session 1, participants in the false denial condition were instructed to deny all of the information when they were interviewed. The following day, participants received a source-monitoring test and were instructed to respond truthfully. What was demonstrated was that participants in the false denial condition exhibited memory impairment for details that they discussed in the first interview. To date, DIF has been replicated in various experiments using different stimuli (i.e., negative and neutral pictures; Otgaar et al., 2016), different memory tasks (recall and recognition; Otgaar et al., 2018), and when participants were instructed to feign memory loss for a crime (Romeo, Otgaar, Smeets, Landström, & Jelicic, 2018).

One issue in previous experiments on the effect of denying on memory is that participants were *explicitly* instructed to falsely deny (e.g., Otgaar et al., 2014; Otgaar et al., 2016; Otgaar et al., 2018; Vieira & Lane, 2013). Of course, in many cases, victims decide themselves whether they will use a certain strategy to cope with an adverse experience. Hence, another important aim of the current experiment was to examine the proportion of participants who choose themselves to use a deceptive strategy. In the current experiment, participants were shown a negative virtual reality scene of an airplane crash that included unpleasant details (e.g., dead bodies on the ground). In order to assess their preferences, all participants were initially allowed to choose a coping strategy from a list of options (i.e., tell the truth, falsely deny, fabricate).

The present study will be the first to examine the memory effect of using a coping strategy following exposure to a traumatic virtual reality (VR) experience. As said before, we were interested in participants’ choices from among the options of truth telling, false denial, or fabrication. We had no specific predictions on how many participants would choose for different coping strategies. However, in case participants were especially choosing to tell the truth, we made sure that in this group, participants would be instructed to falsely deny. Following the MAD framework, our main hypothesis was that the denial-induced forgetting effect would be observed in participants who chose to falsely deny.

## Method

### Participants

Using G*Power (Faul, Erdfelder, Lang, & Buchner, 2007), an a priori power analysis with a power of 0.80 and an anticipated medium effect size (*f* = 0.29) indicated a sample of 95 participants was required. Ninety-five undergraduate students (79 female) from Maastricht University were tested. Their mean age was 21.32 years (*SD* = 2.49; range: 18–35 years). Compensation for both consisted of university credits or a voucher worth €10.00. As a preintervention precaution, the PCL-5 (PTSD checklist) was used to screen participants to ensure that exposure to the experimental stimulus would not compound any preexisting emotional and or psychological problems. The Ethical Committee of the Faculty of Psychology and Neuroscience of Maastricht University granted ethical permission (Reference Number: ERCPN-173_04_11_2016). This study was preregistered and details about the parameters of the design, interview protocols and data can be accessed on the Open Science Framework (OSF): https://osf.io/fgw4s/

### Materials

Categorizations of the true and false detail items that were used in the memory tasks are included in 10.1037/t02622-000 B.

#### Virtual reality (VR) scene

The VR scene was designed in-house by the Department Instrumentation Engineering of the Faculty of Psychology and Neuroscience, Maastricht University. A HTC Vive headset was used and the simulated scene operated on a Dell Precision 5810 computer. The VR scene depicted an airplane crash with dead bodies scattered on the ground. The scene was customized to include several specific visual and auditory details (e.g., sparking wires, a mother crying). Participants’ movement within the VR space was restricted to a 30-centimeter area in order to ensure that their attention was focused on perceiving and processing the scene rather than exploring the VR room (see Appendix Fig. 2). The virtual reality (VR) analogue has been successfully used in previous research as a traumatic stimulus (Dibbets & Schulte-Ostermann, 2015).

#### Baseline memory task

This baseline memory task was self-administered and was a yes/no questionnaire that contained 12 questions (nine true items: e.g., “Was there a body on the ground wearing a red shirt?”; three false items: e.g., “Did the rain begin to fall?”).

#### Memory Task 1

After the baseline memory task another memory task was administered as a structured interview. It contained 12 yes/no questions (e.g., “Did you see a helicopter?”) that also required some participants to restate the question (i.e., false denial: “No, I did not see a helicopter”) or to restate the question and add an additional detail of their choice (i.e., fabrication: “Yes, I saw a helicopter and a fire truck”). They were categorized as follows: five questions related to true presented details that were also included in all memory tasks; three questions related to true details that were also included in the source monitoring task and the memory task that occurred after the source monitoring task; and four questions related to false details that were included only in the source monitoring task and the memory task that occurred after the source monitoring task.

#### Source monitoring task

The source monitoring task was a structured interview that comprised 19 yes/no questions. The questions were categorized as follows: Five questions related to true presented details that were included in all memory tasks; four questions related to true details that were also included in the baseline task; three questions related to true details that were also used in Memory Tasks 1 and 2; four questions related to false details that were also included in Memory Tasks 1 and 2; and three questions related to false details that were also included in the baseline task and Memory Task 2. Each item contained two parts: (a) an interview related question (e.g., “Did the interviewer ask you if there was a body on the ground wearing a red shirt?”) and (b) an event related question (e.g., “Did you see a body on the ground wearing a red shirt?”).

#### Memory Task 2

After the source monitoring task, a second memory task was administered as a structured interview that comprised of 19 yes/no questions. These questions were shuffled restatements of the 19 questions from the source monitoring task and the format of the questions was the same as in the first memory test (after the baseline memory task).

#### PCL-5 (PTSD checklist)

The PCL-5 is a 20-item self-report questionnaire that assesses for symptoms of PTSD based on diagnostic criteria outlined in the *Diagnostic and Statistical Manual of Mental Disorders, Fifth Edition* (DSM-5; American Psychiatric Association, 2013). Participant responses are measured on a 5-point Likert scale from 0 to 4 (e.g., 0 = *not at all* to 4 = *extremely*). Based on findings of the scale’s psychometric properties from two studies, the PCL-5 exhibited strong internal consistency (Cronbach’s *α* = .94), test–retest reliability (*r* = .82), convergent validity (*r*s = .74 to .85), and discriminant validity (*r*s = .31 to .60) (Blevins, Weathers, Davis, Witte, & Domino, 2015). The PCL-5 can be scored in different ways, for example as a total sum, that is, a cumulative score of 33 (out of a total possible score of 80) and above is considered to be indicative of PTSD symptomology (Weathers, Litz, Keane, Palmieri, Marx, & Schnurr, 2013). Alternatively, and as was adopted for this study, scoring can be done via symptom clusters. The 20 questions comprising the PCL-5 are categorized according to symptom clusters as they are outlined in the DSM-5 (American Psychiatric Association, 2013). The questions are represented as follows: 1–5 (Cluster B), 6 and 7 (Cluster C), 8–14 (Cluster E), and 15–20 (Cluster D). Criteria for PTSD symptoms are only considered to be met if a participant responds moderately or higher to one or more questions in Cluster B and Cluster C and to two or more questions in Cluster B and Cluster E (Weathers et al., 2013).^1^ Persons who met the criteria for PTSD were excluded from participating in the study.

#### Additional questionnaires

One questionnaire was administered at the end of the first session, and it assessed participants’ perceptions of the realism of the VR scene. The questionnaire consisted of seven items (e.g., “Did you think the VR clip was traumatic?”). The second questionnaire contained nine items, eight of which were extracted from the Impact of Event Scale–Revised (IES-R; e.g., “Did you have trouble staying asleep?”). The ninth question was a restatement of the sixth item from the first questionnaire (i.e., “Did you think the VR clip was traumatic?”). Both questionnaires were scored on a 5-point Likert scale (i.e., 0 = *not at all* to 4 = *extremely*). These results will not be presented at length in this paper.

### Design and procedure

**Fig. 1.**
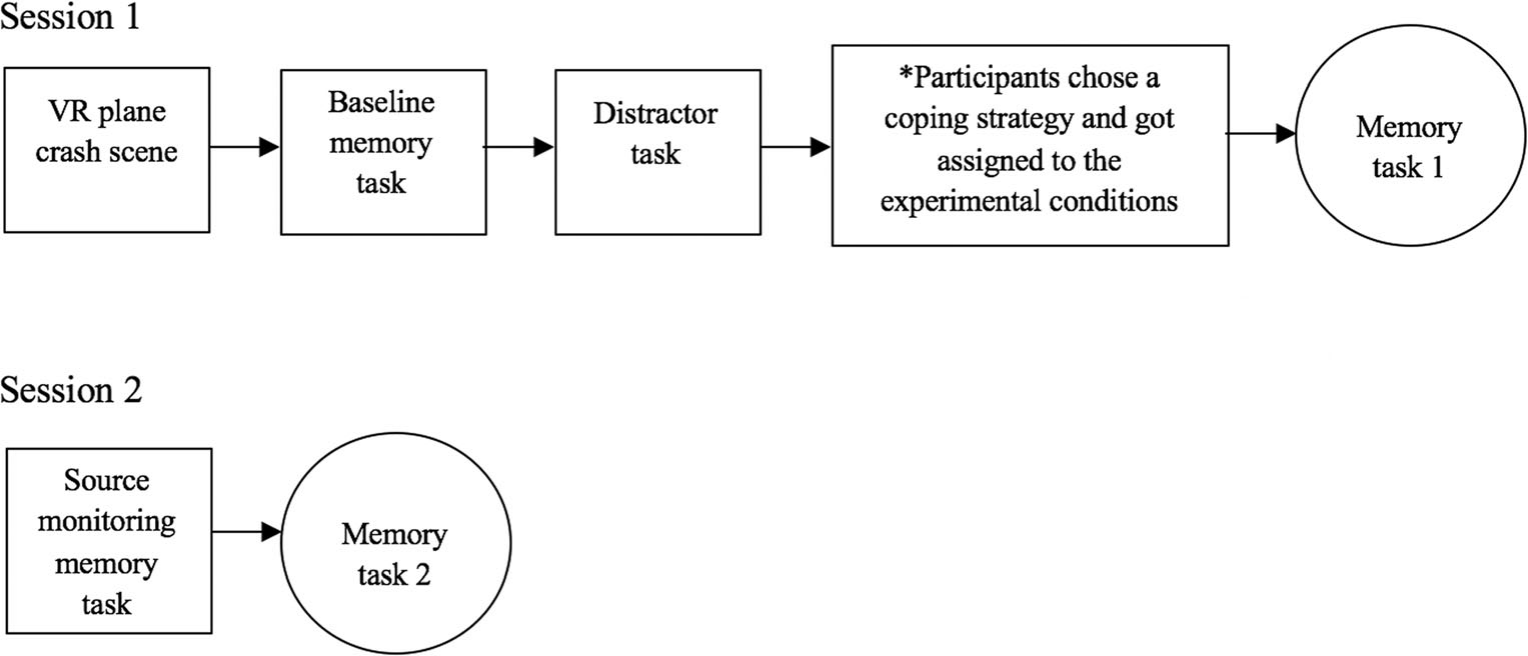
Depiction of the study’s procedure

A between-subjects design was used, and some participants were assigned to different conditions based on their own choices (i.e., truth telling, *n* = 41). Other participants were assigned to conditions randomly a priori to their participation (i.e., directed false denial, *n* = 40; false denial, *n* = 8; fabrication, *n* = 5). Since most participants chose to tell the truth, there was an insufficient number of participants in the false denial and fabrication conditions; therefore, only the data from the truth-telling and the directed false denial conditions were used in the final analyses. A depiction of the study’s procedure can be found in Fig. 1.

In Session 1, participants were shown a scene of a plane crash site in a virtual reality environment for 2 minutes. Next, participants were guided to a separate interview room to complete the baseline memory task, followed by a spot-the-difference distractor task for which participants were allotted 1 minute to identify the discrepancies between two seemingly identical pictures. This was done in order to prevent the extensive rehearsal of information from the VR scene. Following this, participants were given a script that contained definitions of the coping strategies of interest for this study (i.e., truth telling, false denial, and fabrication). They were then told that a strategy was chosen for them through random selection by a computer before the session began, but the interviewer wanted to know what they would choose if given the choice. Through a process of predetermined selection using an Excel table of randomly ordered numbers (i.e., 1 = tell the truth; 2 = falsely deny), the experimenter determined which participants from the truth-telling condition would be forced to falsely deny. A subsample of participants who chose truth telling and all of the participants who chose false denial and fabrication were (falsely) told that their choice was the same as the computerized choice. A subsample of truth tellers were told that “false denial” was selected for them. During the first memory test (after the baseline memory task), truth tellers were instructed to respond honestly to all of the questions. False deniers and directed false deniers were instructed to deny in response to all of the questions (e.g., “No, I did not see a helicopter”); and the fabricators were instructed to respond honestly and add an extra detail (e.g., “Yes, I saw a helicopter and an ambulance”). Session 2 was conducted 1 week later, and a source monitoring task was administered first. Each source monitoring item had two parts. Part A was related to the interview in Session 1 (e.g., “Did the interviewer ask you if you saw a car?”) and Part B was related to what they saw in the VR scene (e.g., “Did you see a car?”). For the last part of Session 2, a second memory test was administered. Finally, participants were asked about their reasons for choosing their strategy in Session 1 (i.e., “What made you choose that strategy in response to seeing the VR scene?”).

### Interrater reliability

In order to understand why participants chose a specific strategy, a data-driven thematic analysis was conducted on responses that were given by participants after Session 2 was concluded (Braun & Clarke, 2006). The first author extracted general themes from the exploratory data and created descriptive codes; this process involved several stages of refinement until main themes were constructed. In a first round, 21 (20% of the combined total of participants from the truth-telling and directed false denial conditions) randomly selected responses were independently rated by the first author and a second rater. Insufficient interrater agreement resulted in meetings to discuss descriptive issues and resolve disagreements. A second round of independent ratings were conducted, which ultimately established the final six themes that were used to rate all of the participants in the truth-telling and directed false denial conditions. Individual kappa values were calculated for each of the six themes that were used to rate participants in the truth-telling and directed false denial conditions (i.e., minimum *K* = 0.37, *p* = 06; maximum *K* = 0.86, *p* < .01). An average kappa value from the six themes showed that there was a substantial level of interrater reliability, *K* = 0.68, *p* = .01. All of the themes and definitions can be found in Appendix Table 3.

## Results

One participant did not attend the second session and was therefore excluded from all analyses, leaving a final sample of 94 participants.

### Baseline memory task

Statistical analyses were conducted between the baseline scores for the truth-telling and directed false denial conditions. A Welch’s independent-samples *t* test showed that the truth-telling (*M* = 7.71, *SD* = 1.06) and directed false denial (*M* = 7.98, *SD* = 0.86) conditions did not statistically differ in their response accuracy for true details, *t*(76.67) = −1.25, *p* = .21, *d* = 0.28, 95% CI [−0.17, 0.73]. For false details, the difference was statistically significant, *t*(51.40) = −2.78, *p* = 0.01, *d* = 0.63, 95% CI [0.17, 1.09] (i.e., truth telling: *M* = 2.78, *SD* = 0.42; directed false denial: *M* = 2.98, *SD* = 0.16).

### Source monitoring task

To examine whether a denial-induced forgetting effect was detected, we assessed the memory performance concerning the interview-related questions of the source monitoring task.

#### Memory for the interview

A Welch’s independent-samples *t* test revealed a statistically significant effect, *t*(79) = 3.95, *p* < .001, *d* = 0.88, 95% CI [0.41, 1.35] for true details that were discussed during the first memory task. Specifically, participants in the directed false denial condition had poorer memory (*M* = 1.55, *SD* = .93) than truth tellers (*M* = 2.29, *SD* = .75). For false details, the difference between the directed false denial (*M* = 1.73, *SD* = 1.22) and truth telling (*M* = 2.66, *SD* = 1.02) condition was also statistically significant, *t*(79) = 3.74, *p* < .001, *d* = 0.83, 95% CI [0.37, 1.29].

#### Memory for the VR scene

We found a statistically significant difference between the two groups for true details that participants reported to have seen in the VR clip, *t*(79) = 2.06, *p* = .04, *d* = 0.45, 95% CI [0, 0.90]). These details were measured in the first memory task but not measured in the baseline questionnaire. Similar to the discussed true details, memory accuracy was lower in the directed false denial group (*M* = 1.33, *SD* = 0.76) than in the truth-telling group (*M* = 1.66, *SD* = 0.69). However, because participants in the directed false denial group were instructed to deny in response to all of the questions in the first memory test, it could not be established whether they did or did not encode those details during exposure to the VR scene. Despite this caveat, it is worth noting that we did not find evidence of analogous memory impairment for true items that were measured in both the baseline questionnaire and the first memory task and true items that were measured in the baseline questionnaire only. Specifically, there was no significant difference between the groups for either of the latter categories, *t*(79) = 0.08, *p* = .94, *d* = 0.01, 95% CI [−0.43, 0.45]; *t*(79) = −1.12, *p* = .26, *d* = 0.25, 95% CI [−0.20, 0.70]). Thus, this difference that was observed between the truth-telling and directed false denial conditions in the first memory task may reflect genuine memory impairment, rather than an artifact.

### Exploratory analyses

#### True items in the baseline and first memory test

Interestingly, we found that no denial-induced forgetting was shown for true items that were presented in the baseline questionnaire first. Specifically, when the directed false denial group (*M* = 4.75, *SD* = 0.93) was instructed to deny these true details during the interview in the first memory test, there was not a statistically significant difference in their level of memory accuracy in the interview in the second session when compared to truth tellers (*M* = 4.88, *SD* = 0.40) when they were interviewed in the second session, *t*(79) = 0.80, *p* = .43, *d* = 0.18).

#### Virtual reality experience

At the end of session one, we assessed participants’ perceptions of the VR scene in terms of realism and traumatic feelings. Five questions were related to perceptions of realism (e.g., “Was the VR world believable?”). To measure realism, each of the five questions was rated on a 5-point Likert scale (i.e., 0 = *not at all* to 4 = *extremely*), and then an average score was calculated. One question was related to perceptions of evoked feelings of trauma (e.g., “Did you think the VR clip was traumatic?”), and this was also rated using the 5-point Likert scale. The final question assessed participants’ history of experiencing a VR environment (e.g., “Have you ever been in a virtual reality environment before?”). Overall, participants perceived the VR scene as moderately realistic (*M* = 2.20, *SD* = 0.59) and moderately traumatic (*M* = 1.73, *SD* = 1.20). Of the total sample, 47 participants reported that they previously experienced a VR environment.

#### Qualitative analyses

Table 1 shows that the most commonly reported theme by the truth-telling group was *alternative disadvantages* (e.g., “Truth telling seemed the most fitting strategy of the three”). Participants in the directed false denial condition also reported *alternative disadvantages* most frequently. The largest difference between the conditions was found in the *psychological outcomes* theme (e.g., “Truth telling is the best way to deal with a traumatic experience”). See Appendix Table 2 for themes and definitions.

**Table 1:** Themes, frequencies, and percentages for participants in the truth-telling and directed false denial conditions

	Truth tellers		Directed false deniers	
Themes	*n*	%	*n*	%
Personal and societal factors	9	21.9	9	22.5
Beneficiary factors	10	24.4	10	25
Alternative disadvantages	12	29.3	11	27.5
Rationale	5	12.2	5	12.5
Psychological outcomes	5	12.2	9	22.5
Miscellaneous	10	24.4	8	20

*Note.* Some participants were assigned ratings for more than one theme. Eighty participants were scored on one or two themes. Only one participant was scored on three themes

## Discussion

The primary aim of this experiment was to determine whether the use of different types of coping strategies would affect memory accuracy. Our most notable finding was that denial undermined memory for what was discussed *and* what was seen by participants. We will now elaborate on the relevance of this finding for theory and practice.

Although participants were afforded the opportunity to choose among three possibilities on how they wanted to cope with the experience of viewing a traumatic virtual reality scene, most participants (*n* = 81) chose to tell the truth. We attributed the preference for truth telling to the following. Participants adopted the role of witnesses and not victims. This lack of self-relevance to the event might make people less willing to use a coping strategy. To address this issue, future research could use a stimulus that is not only realistic but is also one in which the event is self-relevant to the participants (e.g., a VR scene in which participants are attacked). This resulted in two final groups (i.e., truth telling and directed false denial). When looking at the difference in memory performance between these groups, a denial-induced forgetting effect was observed. Specifically, memory for both true and false details that were discussed in the interview in the first session was impaired when participants were re-interviewed one week later. These findings suggest that when people explicitly choose to give honest accounts but then are forced to lie by an external force, their memory for what was discussed can be adversely affected. Our findings are consistent with results from previous studies that examined the effects of false denial on memory (Otgaar et al., 2014; Otgaar et al., 2016; Otgaar et al., 2018; Romeo et al., 2018; Vieira & Lane, 2013).

The current results are line with the MAD framework (Otgaar & Baker, 2018). According to this framework, when people falsely deny, participants are less likely to rehearse the information. Therefore, when people falsely deny, information concerning what is discussed is not processed and stored optimally, resulting in a memory undermining effect. In contrast to previous work on denial-induced forgetting, we focused on which specific details were less likely to be reported and found that false denial did not impair memory for the category of details that were included in both the baseline and first memory task. That is, denial-induced forgetting was only evident for information that was presented for the first time in the first memory task when the directive to deny was given. This implies that false denials only have a specific effect on details when they are discussed at the same moment one is forced to lie. It may be argued that the observed DIF was the result of a lack of processing due to the manner in which the questions were administered and not due to the effortful act of denial. This issue was addressed in previous work by Otgaar et al. (2016). The authors examined whether this explanation of a lack of processing might underlie DIF. They reasoned that if participants did not truly process information in the first session, then memory would also be impaired for details that were only presented in the second session. The idea was that if information for the interview was not processed optimally, then participants would not remember which details were mentioned in the first session and thus deny newly mentioned details in the second session. However, their results showed that false denial rates were actually statistically higher for details that were mentioned during Sessions 1 and 2 (memory was worse) than for newly mentioned details during Session 2. This would be comparable to the finding in the current study that there was not a statistically significant difference for memory in the source monitoring task between the false denial and truth telling groups for true details that were only presented in the baseline task and not Memory Test 1. In fact, this is what we found, that is, *p* = .55; false denial group *M* = 2.93; truth-telling group (*M* = 2.78). Since denial-induced forgetting was not exhibited for these new true details (baseline only), this suggests that during the first memory test, these participants did engage in effortful denial. It is also important to note that prior to the administration of Memory Test 1, participants read a definition list that described the type of behaviour that was reflective of each strategy. This was done to ensure that participants had an idea what a denial entailed. Based on the data that we just presented, we think it is unlikely that participants simply adopted a strategy during the denial.

Our results also revealed some other prominent effects. We found that memory for true details that were actually seen during the virtual reality scene was adversely affected in the (directed) false denial group, although the effect size was rather small (*d* = 0.45). This suggests that false denials might not only impair memory for what discussed, but might also negatively impact memory for the experienced event.

Previous studies on the denial-induced forgetting did not find false denials to adversely affect memory for the stimuli. One reason for why this occurred in the current experiment could be related to the stimuli that were used. Compared with previous studies using pictures (i.e., Otgaar et al., 2016) or videos (i.e., Otgaar et al., 2014; Otgaar et al., 2018), the virtual reality plane crash scene contained highly rich and vivid visual and auditory details. Participants perceived the VR scene to be moderately realistic and traumatic, so perhaps the unpleasant nature of some of the details (e.g., body parts) motivated participants—when falsely denying—to try to not think of what they had seen. The consequence of this is that it might have resulted in the memory undermining effects of false denials. The possibility that this forgetting effect is a false-positive result also cannot be ruled out. Furthermore, to examine the robustness of this memory effect, future research could include a truth-telling control group that only has to tell the truth during the final memory tasks. Such a group would not have an extra opportunity to rehearse information, which is the case in the present experiment. At present, similar to the explanation given for the memory undermining effects of feigned amnesia, we suggest that a lack of rehearsal may have caused the impaired memory effect for the VR event (Christianson & Bylin, 1999; McWilliams; Goodman, Lyons, Newton, & Avila-Mora, 2014; Sun, Punjabi, Greenberg, & Seamon, 2009; Van Oorsouw & Merckelbach, 2004, 2006). More specifically, the root of the adverse effect simulated amnesia on memory has been linked to the obstruction of the rehearsal process. Given that these results are novel in research on false denials, future effort to replicate this finding is recommended. Research along this line should continue to examine the advantages of testing participants by using more realistic stimuli such as virtual reality. Virtual reality provides the advantage of being able to recreate real-life emotionally intense situations that would otherwise be impossible to create (e.g., a plane crash site; Romano, 2005; Visch, Valentijn, Tan, & Molenaar, 2010). Virtual reality also facilitates the complex customization of details in such a way that multiple sensory perceptions and emotions can be tapped into (Waldrop, 2017).

Of note, memory impairment for the VR experience only occurred for true details that were presented to participants in the first memory task. That is, there was no memory impairment for true details that were included in the baseline memory task first and then again in the first memory task, or for true details that were only included in the baseline memory task. This suggests that, for true details that were mentioned in the baseline memory task, this baseline task might have served a protective role and inoculated participants in the (directed) false-denial group, thereby preserving their memory. Again, this is in line with the argument that rehearsal or a lack thereof can cause memory impairment in people who simulate amnesia (Christianson & Bylin, 1999; McWilliams et al., 2014; Sun et al., 2009; Van Oorsouw & Merckelbach, 2004, 2006). So, the baseline memory task facilitated the rehearsal and subsequent encoding of some of the details that were experienced.

To have a rough idea of the reasons participants used to justify their choice of strategy, we also collected qualitative data. The themes that emerged differed across the final groups that were used in our analyses and the excluded false denial and fabrication conditions. In terms of our truth-telling (*n* = 12) and directed false denial groups (*n* = 11), *alternative disadvantages* was the most frequently reported theme. Specifically, participants determined that false denial and fabrication were less advantageous means of coping. For example, the latter strategies were perceived to be “too difficult” or “made remembering harder.” We also found that the largest difference between the truth-telling and directed false denial groups lied in the *psychological outcomes* theme. It seems that being forced to lie caused participants in the directed false denial group to be more aware of the adverse psychological impact of being dishonest about what they observed in the VR scene.

To conclude, this experiment continues along a line of studies that examine the effects of false denial on memory and, more in general, the effects of lying on memory. In order to enhance ecological validity, instead of viewing simple pictures or a video, participants experienced a traumatic VR event containing highly vivid and rich details. To gain some understanding of how memory could be affected, some participants in the experiment who chose to tell the truth were directed to falsely deny. Consistent with previous results, we observed a denial-induced forgetting effect. Denials not only impaired memory for what was discussed, it also negatively affected memory for the experienced event. This implies that lying might even have a more general and damaging role on memory than what has previously been assumed.

## Figures and Tables

**Fig. 2.**
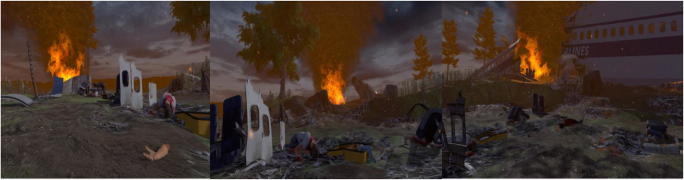
Pictorial Examples from the Virtual Reality Plane Crash Scene

## Appendix 2

**Table 2:** Tasks and question categories

Task	Category
Baseline memory task	5 true (1)
	4 true (2)
	3 false (1)
Memory Task 1	5 true (1)
	3 true (3)
	4 false (2)
Source monitoring task and Memory Task 2	5 true (1)
	4 true (2)
	3 false (1)
	3 true (3)
	4 false (2)

*Note.* These are the categorizations for the number of true and false items that were presented in each task. The bracketed numbers distinguish between different sets of true and false items. For example, there were three distinct sets of true items, that is, (1), (2), and (3). There were two distinct sets of false items, that is, (1) and (2). Note that some sets of items are exclusive to some tasks, and others are repeated

## Appendix 3

**Table:** Table 3

Themes and definitions for the truth telling and directed false denial conditions	
Theme	Definition
Personal and societal factors	The participant based their choice on their personal value system, past behavior, expectations of others, or societal morals
Beneficiary factors	The participant based their choice on who would stand to benefit from the choice (i.e., himself or herself or others)
Alternative disadvantages	The participant based their choice on the perceived disadvantages of the alternative choices (i.e., false denial and fabrication
Rationale	The participant based their choice on rationale and sound reasoning; what seemed to be most sensible
Psychological outcomes	The participant based their choice on the perceived psychological benefits
Miscellaneous	The participant based their choice on a factor that was not captured in the preceding five themes
Themes and definitions for the false denial^1,2,3,4,5^ and fabrication^1,3,4,6^ conditions	
Theme	Definition
^1^Confidence in memory	The participant based their choice on the fact that they were not confident in the memory for the VR scene
^2^External impact	The participant based their choice on the negative ramifications that it could have on others
^3^Psychological outcomes	The participant based their choice on the perceived psychological benefits
^4^Personal habits	The participant based their choice on what was in keeping with their characters; what they would most likely do if they were actually in such a situation
^5^Miscellaneous	The participant based their choice on a factor that was not captured in the preceding four themes
^6^Personal and societal factors	The participant based their choice on their personal value system, past behavior, expectations of others, or societal morals

*Note.* The subscripts indicate which themes are relevant for the false denial and fabrication conditions

## Footnotes

1 See http://www.ptsd.va.gov/professional/assessment/adult-sr/ptsd-checklist.asp
